# Assessing Climate Variability Effects on Dengue Incidence in San Juan, Puerto Rico

**DOI:** 10.3390/ijerph110909409

**Published:** 2014-09-11

**Authors:** Pablo Méndez-Lázaro, Frank E. Muller-Karger, Daniel Otis, Matthew J. McCarthy, Marisol Peña-Orellana

**Affiliations:** 1 Environmental Health Department, Graduate School of Public Health, University of Puerto Rico, Medical Sciences Campus, P.O. Box 365067, San Juan 00936, Puerto Rico; 2 Institute for Marine Remote Sensing, College of Marine Science, University of South Florida, 140 7th Ave. South, St. Petersburg, FL 33701, USA; E-Mails: carib@usf.edu (F.E.M.-K.); dotis@mail.usf.edu (D.O.); mjm8@mail.usf.edu (M.J.M.); 3 Center for Public Health Preparedness, Graduate School of Public Health, University of Puerto Rico, Medical Sciences Campus, P.O. Box 365067, San Juan 00936, Puerto Rico; E-Mail: marisol.pena@upr.edu

**Keywords:** climate change, extreme weather, dengue transmission, San Juan, Puerto Rico

## Abstract

We test the hypothesis that climate and environmental conditions are becoming favorable for dengue transmission in San Juan, Puerto Rico. Sea Level Pressure (SLP), Mean Sea Level (MSL), Wind, Sea Surface Temperature (SST), Air Surface Temperature (AST), Rainfall, and confirmed dengue cases were analyzed. We evaluated the dengue incidence and environmental data with Principal Component Analysis, Pearson correlation coefficient, Mann-Kendall trend test and logistic regressions. Results indicated that dry days are increasing and wet days are decreasing. MSL is increasing, posing higher risk of dengue as the perimeter of the San Juan Bay estuary expands and shorelines move inland. Warming is evident with both SST and AST. Maximum and minimum air surface temperature extremes have increased. Between 1992 and 2011, dengue transmission increased by a factor of 3.4 (95% CI: 1.9–6.1) for each 1 °C increase in SST. For the period 2007–2011 alone, dengue incidence reached a factor of 5.2 (95% CI: 1.9–13.9) for each 1 °C increase in SST. Teenagers are consistently the age group that suffers the most infections in San Juan. Results help understand possible impacts of different climate change scenarios in planning for social adaptation and public health interventions.

## 1. Introduction

Relationships between vector-borne infectious disease and the environmental conditions of a particular region are well documented [1,2,3,4,5,6,7]. Meanwhile, much of modern society now recognizes that it has been subjected to changing environmental conditions since the last half of the 19th century [8]. Around the world, many parameters including air and ocean temperatures, wind, precipitation, and associated river discharge are showing secular trends as well as increased variability from year to year [9,10,11,12,13,14]. Such shifts and associated extreme events are likely to have an influence on the epidemiology of vector-borne diseases [3].

Rising average temperatures can lead to the expansion of the geographic range of many vectors, to decreasing extrinsic incubation periods of many pathogens, and to an increased rate of contact of mosquitoes (such as *Aedes aegypti* and *Aedes albopictus*) with prey including humans [4,5]. Mean Sea Level also influences the density of salinity-tolerant vector mosquitoes along the coast. Sea level rise could lead to the adaptation of freshwater vectors to breed in brackish and saline waters [15]. Numerical simulations point to an acceleration of the hydrologic cycle in a warmer climate, a phenomenon which may in part explain the higher frequency of extreme events observed over the course of the past few decades [8]. The impacts of these changes on humans are compounded by demographic, social, and economic factors [16,17]. Thus, it is necessary to examine how environmental patterns may be shifting in areas that may be increasingly prone to vector-borne infectious diseases. Johansson *et al.* [18] and Moore *et al.* [1] present evidence that the abundance and the transmission potential of *Aedes aegypti* in Puerto Rico are influenced by temperature and precipitation. Some investigators have suggested that regional climate conditions and sea level rise can also influence dengue outbreaks [19,20]. Significant correlations have already been found between sea surface temperature and dengue cases in coastal areas of Mexico and in New Caledonia [4,7,21].

In this paper, we examine the time history of confirmed dengue fever cases in the city of San Juan, Puerto Rico, as an urban example of a tropical island environment that is experiencing rapid environmental and socio-economic changes. At present, there is no effective vaccine or therapy to counter the symptoms of dengue. Much effort is thus placed on disease prevention, including vector control strategies and health education. However, such efforts have had mixed success [22,23]. In Puerto Rico, large epidemics have recurred every 3–5 years (epidemics are defined as three or more suspected dengue infections reported per 1000 individuals for two consecutive weeks) [22]. Among the most recent are the epidemics of 2007, when 10,576 suspected cases were reported, and of 2010 with 26,776 suspected cases [24]. These events highlight the need to define areas at risk, and understanding factors that affect timing of the disease to plan for better and more effective control interventions.

We test the hypothesis that conditions are becoming increasingly favorable for dengue transmission in San Juan, Puerto Rico, and that this pattern is representative of potential expansion of dengue virus on the island. We address questions of vulnerability, exposure, and adaptation to effects of climate change. Our results help to define better strategies for improving public health interventions for dengue in Puerto Rico.

## 2. Study Area

**Figure 1:**
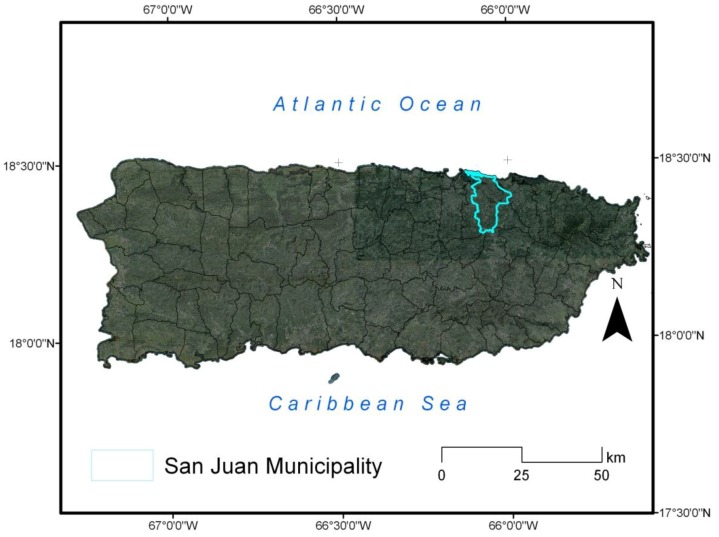
Puerto Rico and location of the municipality of San Juan.

Puerto Rico is an island located in the northern-central Caribbean Sea (17.92°N–18.52°N, 65.62°W–67.28°W). The municipality of San Juan, located in the northeast sector of the island, is the capital in Puerto Rico and an urban coastal area (Figure 1). San Juan has a subtropical humid climate; with an annual average rainfall of ~1800 mm. Easterly trade winds prevail most of the year over the island, with local winds influenced by the diurnal heating cycle. A sea breeze is observed along the north, south, and west coastal sections [25]. Average air temperatures range from 22–28 °C [9,26].

## 3. Methods

### 3.1. Data Collection

A long time-series of observations are necessary to evaluate variation and trends in oceanographic and meteorological conditions over scales ranging from daily to multidecadal [27,28]. To assess the relationship between environmental parameters and the frequency of occurrence of dengue fever in the municipality of San Juan, we examined various daily and monthly data for the variables listed in Table 1. These environmental parameters are important in defining the most suitable conditions and the habitat of the vectors that transmit the dengue fever virus [1,2,3,4,5]. Specifically, we examined daily surface air temperature (maximum and minimum), precipitation, sea level pressure, and wind speed data. These observations were obtained from the NOAA-National Climatic Data Center [29]. Since coastal flooding and brackish waters also represent potential habitat for the mosquitoes, we processed hourly mean sea level (MSL) observations (data for San Juan station obtained from the University of Hawaii Sea Level Center/National Oceanographic Data Center, Honolulu, HI, USA. The MSL data were processed to monthly mean values to minimize the effect of tides on an analysis of longer-term variations. Monthly Mean Sea Surface Temperature (SST) was computed based on daily satellite-based observations using the NOAA Advanced Very High Resolution (AVHRR) Pathfinder SST product (version 5.2), using a set of 4 × 4 pixels (*i.e.*, an area of approximately 256 km^2^ located immediately off the coast of San Juan. Numbers of confirmed dengue fever cases were obtained from the Dengue Branch of the Center for Disease Control and Prevention (CDC) in San Juan. These records were assembled by the CDC and the Puerto Rico Department of Health (PRDH) Passive Dengue Surveillance System (PDSS).

**Table 1:** Database and periods studied.

Variable	Period	Time Step
Dengue Cases	1992–2011	Daily
Rainfall	1899–2011	Daily
Air Surface Temperature	1899–2011	Daily
Sea Surface Temperature	1981–2012	Monthly
Sea Level Pressure	1978–2012	Daily
Wind Speed	1978–2012	Daily
Mean Sea Level	1978–2012	Daily

The International Expert Team on Climate Change Detection and Indices (ETCCDI) recommend 27 indices for monitoring changes in extreme environmental conditions [13,30,31]. We derived seven of the ETCCDI indices based on temperature and six indices based on rainfall for this study (Table 2). Frequency of extreme occurrences in MSL (non-tidal), Wind, SLP, SST, and dengue were derived as maxima and percentiles [32,33,34]. Monthly and annual averages, amplitudes, and anomalies of these variables were also examined.

### 3.2. Data Analysis

We examined variability and trends in a number of parameters. Principal component analysis (PCA) was used to summarize possible relationships between all variables in years where all data were available (1992–2011) [35,36,37]. The PCA helps to determine the amount of the total variance that can be explained by combinations of particular factors. Logistic regression was used to explore the threshold value of the predictors established by the PCA and bivariate analysis [35,38,39]. To examine whether there was an association between the number of dengue cases and the various environmental parameters, we calculated cross-correlation coefficients. The Pearson correlation coefficient was used to evaluate the strength of such correlation. The Mann-Kendall (MK) test was used to test for the significance of time series trends [40,41,42]. The null hypothesis (H0) meant that there was no trend in the series.

**Table 2:** The Extreme Temperature and Precipitation Indices.

ID	Indicator Name	Indicator Dentitions	Units
TN10p	Cool nights	Percentage of time when daily min temperature < 10th percentile	%
TX10p	Cool days	Percentage of time when daily max temperature < 10th percentile	%
TN90p	Warm nights	Percentage of time when daily min temperature > 90th Percentile	%
TX90p	Warm days	Percentage of time when daily max temperature > 90th percentile	%
WSDI	Warm spell duration indicator	Annual count when at least six consecutive days of max temperature > 90th percentile	days
CSDIN	Cold spell duration indicator	Annual count when at least six consecutive days of min temperature < 10th percentile	days
CSDIX	Cold spell duration indicator	Annual count when at least six consecutive days of max temperature < 10th percentile	days
PRCPTOT	Annual total wet-day precipitation	Annual total precipitation from days ≥ 1 mm	mm
R10	Number of heavy precipitation days	Annual count when precipitation ≥ 10 mm	days
R20	Number of very heavy precipitation days	Annual count when precipitation ≥ 20 mm	days
CDD	Consecutive dry days	Maximum number of consecutive days when precipitation < 1 mm	days
CWD	Consecutive wet days	Maximum number of consecutive days when precipitation ≥ 1 mm	days
R95p	Very wet days	Annual total precipitation from days > 95th percentile	mm
R99p	Extremely wet days	Annual total precipitation from days > 99th percentile	mm

## 4. Results and Discussion

### 4.1. Oceanographic and Meteorological Trends (1899–2011)

**Figure 2:**
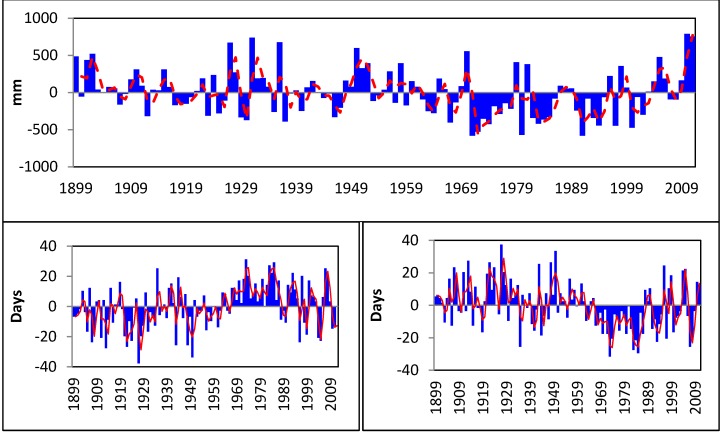
Upper Panel: Annual rainfall anomaly (blue bars) and moving average (red broken line). Lower Left: Anomalies for dry days (blue bars) and moving average (red line); Lower Right: Anomalies for wet days (blue bars) and moving average (red line); 1899–2011.

Precipitation in San Juan since 1899 (Figure 2) shows marked wet (1948–1952, 2003–2006; 2009–2011) and dry periods (e.g., 1971–1977, 1982–1984, 1990–1995). A slight (not significant) downward trend in precipitation (−0.95 mm/year) is observed over the 1899–2011 period, primarily driven by the higher number of dry years observed in this region between about 1950 and the late 1990s. There has been a marked rise in positive annual and daily rainfall anomalies since the 1990s (Figure 2). Numbers of dry days are increasing (especially in January, February, March and June) while wet days show a significant decreasing trend (Figure 2). These results agree with regional trends and climate projections for the Caribbean Region [13].

**Figure 3:**
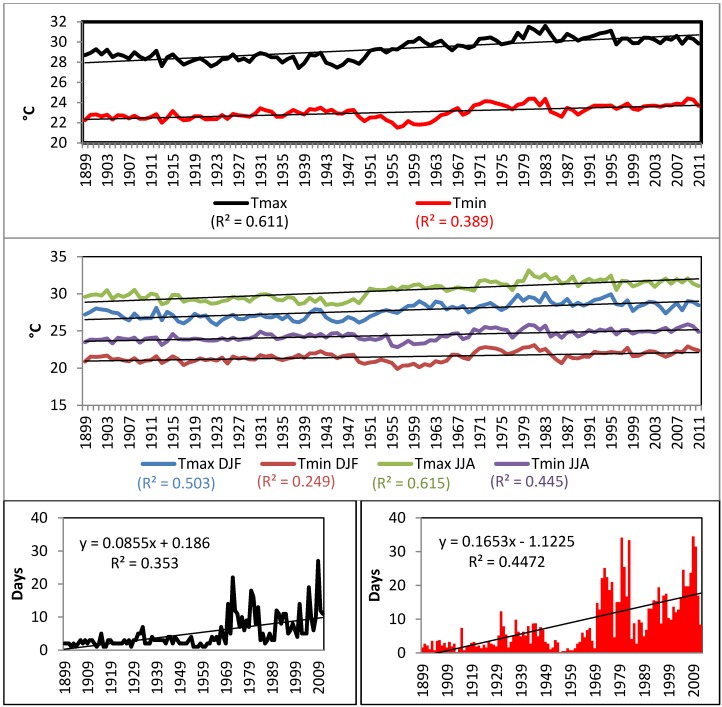
Upper Panel: Time series of annual average minimum and average maximum air surface temperature. Middle: Seasonal Trends in annual average minimum and maximum air temperature. DJF indicates December-January-February and JJA indicates June-July-August. Lower Left: Consecutive days > 90th percentile Tmin (25 °C). Lower Right: TN90p (San Juan 1899–2011).

A significant positive trend was observed in annual mean maximum and minimum air temperatures, as well as in seasonal minimum and maximum temperature extremes (Figure 3). The entire Caribbean region warmed significantly over the period 1961–2009 [13,43]. The annual percentage of warm days and nights, analyzed through the TX90p and TN90p indices, has significantly increased. Our results are also consistent with trends observed over parts of northern South America, where nighttime (minimum) temperature indices show the largest rates of warming [44]. Daytime (maximum) temperature indices also show warming over much of South America, but at lower rates.

There was a significant increasing trend with the number of days per year when SLP records were <10th percentile. The minimum values for SLP occurred between August and November. These are months of high tropical cyclone activity in the Caribbean Sea and the North Atlantic. Maximum SLP showed a significant downward trend, suggesting that SLP amplitude is diminishing over time (Table 3). This is consistent with the displacement of the Intertropical Convergence Zone (ITCZ) away from the equator toward the northern subtropics and possibly the effects of a stronger subtropical jet on the northern Caribbean Sea [45,46,47,48].

**Figure 4:**
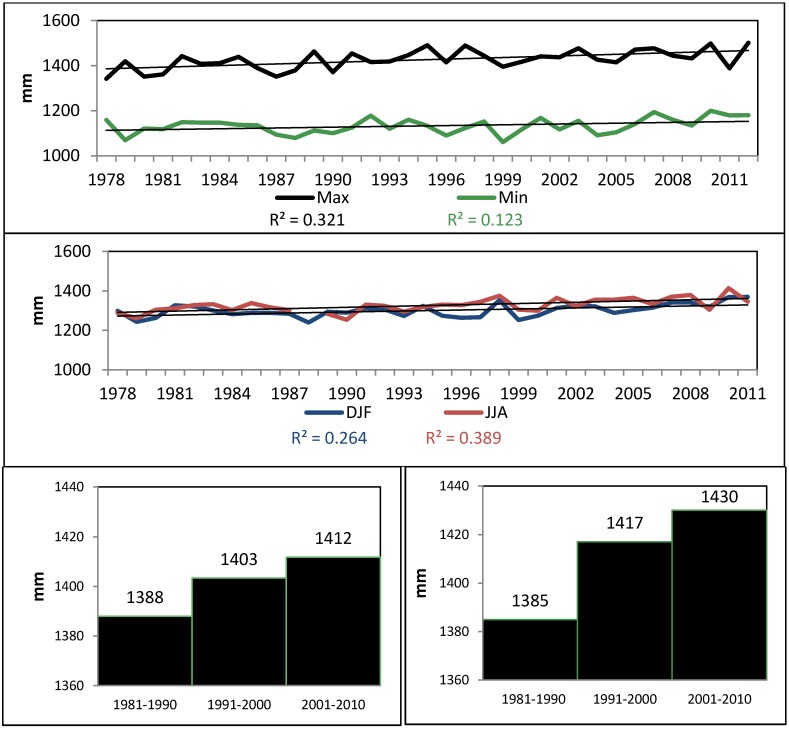
Upper Panel: Annual Maximum and Minimum Mean Sea Level. Middle: Seasonal Mean Sea Level (DJF-December, January and February; JJA-June, July and August). Lower Left: Maximum Annual Mean Sea Level trends. Lower Right: Mean Sea Level annual 90th percentile per decade.

Higher MSL values in San Juan Bay occurred between August and October. The timing of the extreme (maximum annual MSL) also occurred during this period. Sea-level extremes (90th percentile) rose by up to 1.5 mm/year. in the San Juan Bay area (Figure 4), in line with previously estimated Sea Level Rise from NOAA (1.65 mm/year.). Clearly, higher sea level extremes occur superimposed on a gradually increasing mean sea level [49].

**Figure 5:**
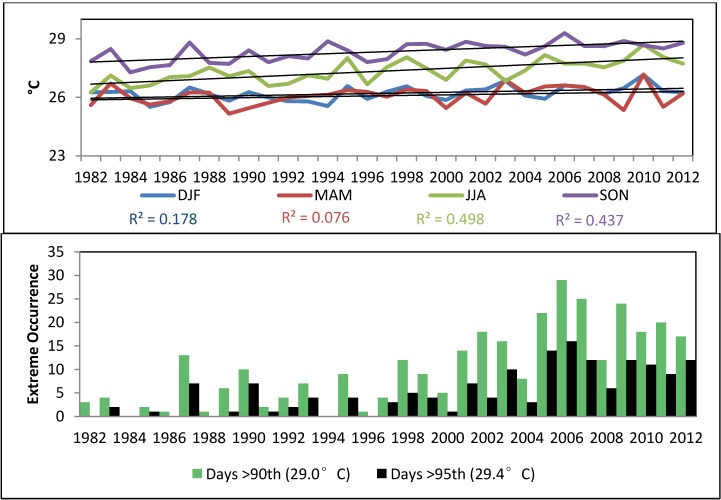
Top Panel: Seasonal Sea Surface Temperature trends (AVHRR Pathfinder v5.2 SST, extracted in a 4x4 km^2^ area in the Atlantic Ocean immediately off San Juan 1981–2012). Lower Panel: Number of days showing extremely high SST (above the 90th percentile: 29 °C, and 95th percentile: 29.4 °C).

SST values are also increasing in the Caribbean Sea [43], especially between June and December. Extreme high SSTs in the period 1981–2012 were normally more frequent in September, October, and November. The 90th percentile showed an upward trend in all seasons, with larger trends in September, October, and November (+0.84 °C) compared to winter and spring (Figure 5).

Average wind speed decreased between 1978 and 2012, while wind direction amplitude increased. Wind speed was usually lower when the wind direction turned from the south. As Maximum Air Surface Temperature and Sea Surface Temperature increased, Minimum Air Surface Temperature in San Juan decreased, primarily at night. The concurrent slightly long-term trend of decreasing precipitation led us to conclude that this night-time cooling was likely due to an increase in the occurrence of clear skies along the north coast. In the San Juan area, southerly winds lead to a decrease in moisture in the lee of the mountains of the Cordillera Central (*i.e.*, the northern coast). When winds blow from the Northeast, higher humidity typically occurs along the north side of the island.

**Table 3:** Ocean and meteorological Mann-Kendall trend analysis. Positive “S” indicates a positive trend. Negative “S” values indicate negative trends. If *p* < 0.05, the slope is significantly different from zero.

ID	S	*p*-value (Two-Tailed)	Test Interpretation
CWD	−195	0.63	Accept H0
CDD	516	0.20	Accept H0
#Days > 1 mm	**−1382**	**0.001**	**Reject H0**
Max Consecutive days > 90th (13 mm)	−169	0.66	Accept H0
Max Consecutive days R10 mm	66	0.86	Accept H0
#Days < 1 mm	**1403**	**0.001**	**Reject H0**
R10	−676	0.09	Accept H0
R20	−407	0.31	Accept H0
Max Consecutive days > 95th (22.1 mm)	−412	0.26	Accept H0
Max Consecutive days> R20 mm	−119	0.75	Accept H0
Max Consecutive days > 99th (49.8 mm)	−74	0.82	Accept H0
#Days > 99th (49.8 mm)	168	0.67	Accept H0
#Days > 95th (22.1 mm)	−544	0.18	Accept H0
TN10p	**−1167**	**0.002**	**Reject H0**
TX10p	**−1313**	**0.001**	**Reject H0**
TN90p	**3102**	**0.0001**	**Reject H0**
TX90p	**3535**	**0.0001**	**Reject H0**
WSDI	**3109**	**0.0001**	**Reject H0**
CSDIX	**−1263**	**0.001**	**Reject H0**
CSDIN	**−1124**	**0.003**	**Reject H0**
SLP Annual Max	**−310**	**0.0001**	**Reject H0**
SLP Annual Min	36	0.591	Accept H0
SLP Annual Average	**−184**	**0.004**	**Reject H0**
SLP #Days < 10th percentile	**146**	**0.014**	**Reject H0**
SLP Monthly Max	**−1816**	**0.0001**	**Reject H0**
MSL Max	**242**	**0.0001**	**Reject H0**
MSL Min	**212**	**0.002**	**Reject H0**
MSL Average	**225**	**0.001**	**Reject H0**
SST Annual Max	**219**	**0.0001**	**Reject H0**
SST Annual Min	**99**	**0.09**	Accept H0
Wind Speed Annual Average	**−81**	**0.238**	Accept H0
Wind Speed Annual Max	**−255**	**0.0001**	**Reject H0**

### 4.2. Association between Dengue Fever and Ocean- and Meteorological Change (1992–2011)

**Figure 6:**
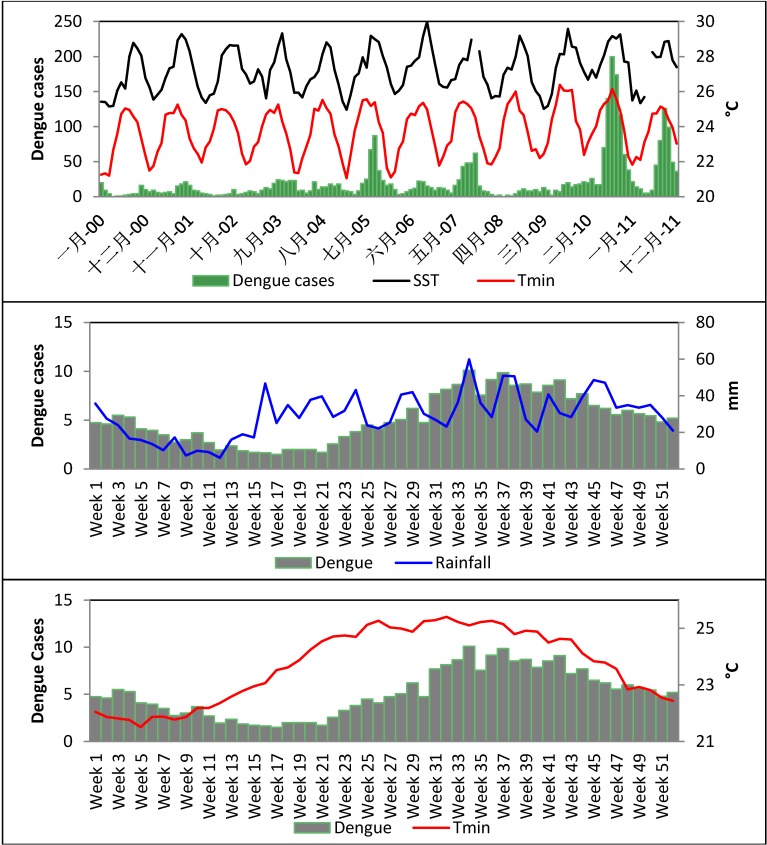
Upper: Monthly sea surface temperature (SST), minimum air surface temperature (Tmin) and monthly dengue cases (2000–2011). Middle: Weekly rainfall and dengue. Lower: Minimum air surface temperature and weekly dengue cases.

Dengue records collected since 1992 show that this disease has a seasonal periodicity in Puerto Rico (Figure 6). Three phases are normally observed during each period. A pre-epidemic phase occurs between February and May (*i.e.*, weeks 10–20), with rising dengue cases (Figure 6). This is a time when seasonal air and ocean temperatures start to rise, when there is relatively abundant precipitation due to passage of winter cold fronts, and there is an increasing numbers of mosquitoes. Epidemics are then observed from about June (week 23) to October (week 40), when temperatures have risen the fastest and reach maxima, and until after maxima in temperature and precipitation are reached. The post-epidemic phase, when dengue fever cases decrease, occurs from November to January. The phases we identify here are slightly out of phase with those described previously [6].

The Pearson correlation analysis showed that dengue cases were significantly associated with at least 5 variables (SST, AST, Rainfall, MSL, and SLP). The strongest association was with SST and AST. Correlation between SST and dengue cases increased significantly if data were separated in five year periods (1992–1996, *r* = 0.04; 1997–2001, *r* = 0.26; 2002–2006, *r* = 0.45; 2007–2011, *r* = 0.56). Two of the largest and longest epidemics in Puerto Rico history occurred in the last period, *i.e.*, in 2007 and 2010. The PCA shows that four environmental factors together explained 72% of the variance in the dengue data. Sea Surface Temperature dominated the first PCA factor (0.90), followed by Minimum Air Surface Temperature (0.79) and MSL (0.79). Mean Sea Level, however, is highly correlated with SST (*r* = 0.74), as is Minimum Air Surface Temperature (*r* = 0.53). Thus, autocorrelation issues inflate these statistics (Table 4).

Warmer SST and more variable wind speed and direction in Puerto Rico correspond to a more northerly position of the ITCZ. In general, warm years registered in SST are associated with wet years (especially in the late rainy season when rainfall was above normal e.g., 2003–2006; 2009–2011) while cooler episodes are associated with drier periods (especially in the early rainy season when rainfall was below normal e.g., 1982–1986; 1990–1995). Previous studies have not found an association between these parameters, dengue, and episodes of El Niño South Oscillation (ENSO) [18,21]. We did not find a relationship with ENSO either.

Our results also show a link between dengue fever, rainfall, and changes in minimum air surface temperature. While warm nights (TN90p) and nocturnal warm spells (consecutive days > 25 °C) seemed to have a small role to play in dengue occurrence (*r* = 0.33), maximum air surface temperature patterns didn’t seem to have a strong influence on dengue. Higher maximum temperatures tend to decline egg hatching rates while higher minimum daytime temperatures are likely leading to shorter virus incubation periods in the vector, shorter viral development rates, and shorter mosquito larvae development times [6,50,51]. Many researchers suggest that both *Aedes aegypti* and *Aedes albopictus* have excellent adaptation skills regarding rising temperatures and extreme conditions [52]. Other scientists have found that higher temperatures produce significantly smaller adults since as temperatures increase, the mosquito development time is reduced [51,52].

**Table 4:** Monthly correlation matrix Pearson. 1992–2011.

Periods	Variables	Dengue Cases	SST	SLP	Rainfall	Tmax	Tmin	MSL
1992–2011	Dengue Cases	**1**	**0.36**	**−0.31**	0.22	0.16	0.25	**0.33**
	SST	**0.36**	**1**	**−0.63**	**0.48**	**0.63**	**0.72**	**0.74**
	SLP	**−0.31**	**−0.63**	**1**	**−0.40**	**−0.36**	**−0.41**	**−0.59**
	Rainfall	0.22	**0.48**	**−0.39**	**1**	0.19	**0.38**	0.29
	Tmax	0.16	**0.63**	**−0.35**	0.19	**1**	**0.90**	**0.47**
	Tmin	0.25	**0.72**	**−0.41**	**0.38**	**0.90**	**1**	**0.53**
	MSL	**0.33**	**0.74**	**−0.59**	0.29	**0.47**	**0.53**	**1**

Note: Values in bold are different from 0 with a significance level alpha = 0.05.

The correlation between SST and dengue incidence is high for San Juan. For example, monthly dengue transmission rates between 2000 and 2011 were 3.4 times higher (95% CI: 1.9–6.1) for each 1 °C increase in SST and 2.2 higher (95% CI: 1.3–3.5) for each 1 °C increase in Minimum Air Surface Temperature. These factors were further accelerated in the 2007-2011 period, with monthly dengue transmission being a factor of 5.2 higher (95% CI: 1.9–13.9) for 1 °C increases in Sea Surface Temperature.

The number of days per year when precipitation is >10 mm/24 h also leads to higher number of consecutive days with dengue transmission (*r* = 0.35). No significant results were obtained with R20 (>20 mm/24 h), suggesting that excessive rainfall events have no incremental effect on dengue cases. Nevertheless, the number of wet days a year is a predictor for dengue. When more consecutive wet days occurred in a year, dengue incidence increased. Higher rainfall leads to an increase in breeding sites of the mosquito vector [50], which would contribute to the increase in dengue occurrence.

The diversity and distribution of mosquitoes in Puerto Rico have not been studied extensively, and background literature on the ecology of these vectors is limited [53]. The observation of *Aedes albopictus* in San Juan, is recent, which suggests that this species is a relatively recent introduction to the island. As sea level rises, the boundaries of the estuary of San Juan Bay are moving inland. The strong correlation between dengue, MSL, and the high incidence of mosquitoes (now both *Aedes aegypti and Aedes albopictus*) in brackish environments [15,54,55,56], also suggest that the risk of dengue cases is increasing as the perimeter of the estuary expands. Therefore, even though there is still lack of concrete evidence that vectors are proliferating in brackish waters on the island, these findings encourage further research on vector ecology. There exist a substantial need in San Juan to study these vectors within the estuary’s boundaries given the possibility that brackish water-adapted *Aedes aegypti* and *Aedes albopictus*, may play an up till now unrecognized role in transmitting dengue and chikungunya in coastal urban areas [54]. Chikungunya virus was also detected in San Juan-Puerto Rico for the first time in 2014, with over 200 cases reported island-wide by the time of this writing in mid-2014 alone [57]. This presents a new and clear threat to public health concerning vector-borne diseases.

**Figure 7:**
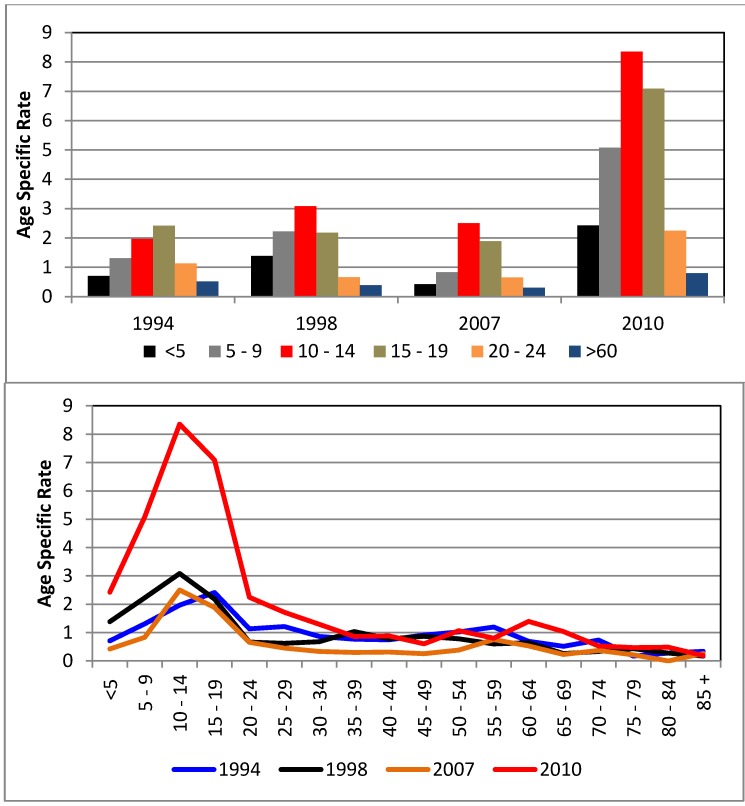
Comparison of age-specific dengue incidence rates per 1000 inhabitants in epidemic years (1994, 1998, 2007, and 2010) in San Juan. Upper Panel: Age-specific dengue incidence rates (age groups <24 years old and >60 years old). Lower Panel: Age-specific dengue incidence rates (all age groups).

There have been several years with epidemic dengue outbreaks in Puerto Rico, specifically 1994, 1998, 2007 and 2010 [22,23,24,58,59,60]. In San Juan, 65% of the confirmed cases in 1994 were patients younger than 30 years old (Figure 7). The most affected age-group in 1998 were individuals 10–14 years old (3.1 cases per 1000 individuals). In 2007, a total of 17,000 cases were reported island-wide, with an incidence rate of 4.8/1000 individuals, predominantly in the 10–19 age group [58]. In many dengue-prone countries, young children bear the greatest burden of the disease; yet, a gradual shift in peak attack rate towards older age groups has also been noted [58]. In Puerto Rico, and especially in San Juan, teenagers still are consistently the age group that suffers the most infections.

Of particular concern in Puerto Rico were the high numbers of patients observed in 2010 (more than 12,000 confirmed dengue cases with 40 deaths) [24,58,59]. The 10–14 age group accounted for 23% of incidences (the age-specific incidence rate was 8.4/1000 inhabitants) followed by the 15–19 age group (7.1/1000 inhabitants). The third group was comprised of the 5–9 age group (5.1/1000 inhabitants), with 13% of the confirmed cases [24]. This has been the deadliest outbreak registered in Puerto Rico and in the United States thus far [24,60].

All four dengue virus serotypes occurred during each of these epidemics, but usually one serotype seems to be dominant over the others. Exposure and infection by one dengue serotype leads to lifelong serotype-specific immunity and short term cross protection against another serotype [59]. For example, in 1994 and 1998, dengue virus serotype 2 dominated in San Juan. During the 1998 dengue epidemic, type 3 virus also occurred frequently in Puerto Rico after an absence of 20 years [24]. In the 2007 epidemic, nearly 70% of the cases in San Juan were confirmed as virus type 3. During the 2010 outbreak, virus 1 dominated, followed by virus 4, with 48% and 20% respectively.

2010 was a year of a pan-American dengue outbreak. Over 1.7 million cases were reported across America and the Caribbean [61,62]. Two main factors may have enhanced virus transmission in the northern Caribbean Sea, one may have been low population immunity against the circulating serotype (types 1 and 4), as in the French West Indies [62], and climatic conditions. 2010 was one of the warmest years since 1850 in San Juan [63] and in the Caribbean Sea in general (SST 1 °C above the average between 1978–2012). It was also the wettest year on record since 1899 (788 mm above the average 1899–2011). 2010 also showed the second highest Mean Sea Level since 1978 (the maximum in our time series records was in 2012), the second highest mean Air Surface Temperature since 1899 (the maximum was in 2009), and the lowest monthly average Sea Level Pressure (1978–2012).

The oceanographic and climate conditions or the serotype profile are clearly not the only factors that define the temporal patterns or pathological ecology of dengue [64]. Other important factors include immunity and mobility of the population, socio-economic factors (inequality and poverty), public policy, implementation of surveillance systems, dengue control programs, deficient septic tanks conditions, among others, also play a role [35,50,38,64,65,66,67]. Our research primarily emphasizes the need for interdisciplinary collaboration to incorporate assessments of temporal patterns of dengue transmission, environmental information, and of climate change projections into the design of climate change adaptation programs, along with social data.

## 5. Conclusions

Our results show significant correlations between dengue fever occurrence in San Juan, Puerto Rico and a number of environmental indicators of climate change. While it is difficult to explain causality, these variables are known to each have various effects on both vector and dengue virus. These are similar variables that affect the ecology of the vector of new pathogens, such as the chikungunya virus. Clearly, environmental factors and climate conditions enhance or diminish the risk posed by social and economic factors such as urban planning, degree of sanitation, infrastructure that may affect mosquito habitat, and behavior of particular age groups.

It is also important to evaluate short-term variability in the context of longer-term trends. As northern Puerto Rico experiences a long-term decrease in annual precipitation, short term increase in precipitation as observed since 2009 likely plays a role in the higher incidence of dengue fever observed in the municipality of San Juan. The occurrence of dengue fever shows strong correlation with various other environmental indicators of increased favorable habitat for the vector. Even though maximum and minimum air surface temperature extremes have increased over time in the region, dengue cases were more frequent during periods when more days with higher minimum air surface temperatures were observed. Previous studies suggest that consecutive days with higher temperature affect egg hatching, virus incubation, and mosquito larvae development. There was no correlation with maxima surface air temperature; however, there was a significant statistical relationship with SST. This reflects the tight coupling between the ecology of the island and oceanic conditions.

MSL was another variable related to dengue occurrence. Since historical MSL records show a clear increasing trend, this highlights the potential risk of new habitat created for the vector as the perimeter of San Juan Bay moves inland. Since MSL maxima are seasonal, coastal areas will be prone to increased flooding during the period which already shows the highest dengue incidences every year.

This research shows the need for inter-disciplinary collaboration and to incorporate assessments of environmental changes over large temporal and spatial scales in efforts to understand patterns of dengue transmission. This effort puts the impacts of climate, demographic, and social change in context. The knowledge gained through such studies helps focus efforts in vector control, given the likely continuing changes expected in SST, AST and MSL as a result of climate change over the next few decades. This knowledge needs to be linked to demographic and socio-economic patterns to help in defining mitigating strategies. Additional research is needed to help understand patterns in other municipalities of Puerto Rico, and in other tropical islands and mainland locations.
